# IN TIME: THE VALUE AND GLOBAL IMPLICATIONSOF NEWBORN SCREENING FORSEVERE COMBINED IMMUNODEFICIENCY

**DOI:** 10.1590/1984-0462/;2018;36;4;00020

**Published:** 2018

**Authors:** Cristina Meehan, Carmem Bonfim, Joseph F. Dasso, Beatriz Tavares Costa-Carvalho, Antonio Condino-Neto, Jolan Walter

**Affiliations:** a Division of Allergy and Immunology, Children’s Research Institute, University of South Florida, St. Petersburg, FL, United States.; b Clinics Hospital, Universidade Federal do Paraná, Curitiba, PR, Brazil; c Department of Biology, University of Tampa, Tampa, FL, United States.; d Division of Allergy, Clinical Immunology and Rheumatology, Department of Pediatrics, Universidade Federal de São Paulo, São Paulo, SP, Brazil.; e Department of Immunology, Institute of Biomedical Sciences, Universidade de São Paulo, São Paulo, SP, Brazil.; f Division of Allergy and Immunology, Johns Hopkins All Children’s Hospital, St. Petersburg, FL, United States.; g Division of Pediatric Allergy and Immunology, Massachusetts General Hospital, Boston, MA, United States.

## SEVERE COMBINED IMMUNODEFICIENCY (SCID)

Severe combined immunodeficiency (SCID) is recognized as a global pediatric emergency that manifests early in infancy.^1^ Inthe absence of adaptive cellular and humoral immune response, infants with SCID are prone to life threatening infections around 4-6 months of age, as they lose protective maternal antibodies. Therefore, there is a narrow window of opportunity for early detection of infants with SCID during the asymptomatic period around birth. Newborn screening (NBS) is an essential solution for timely recognition and treatment of this otherwise fatal pediatric disease.

Specifically, infants with SCID are highly susceptible to a broad spectrum of bacterial, fungal and viral infections. Inaddition to typical and opportunistic infections, live attenuated vaccine agents including Bacillus Calmette-Guérin (BCG) for tuberculosis, the oral poliovirus and rotavirus vaccines can result in severe complications including disseminated disease.^2^ ^,^ ^3^ ^,^ ^4^ Therefore, it is imperative to perform NBS for SCID before live vaccines are administered, so patients at risk can be identified and the potentially harmful routine live vaccinations can be avoided for this vulnerable patient population.

**Figure 1:**
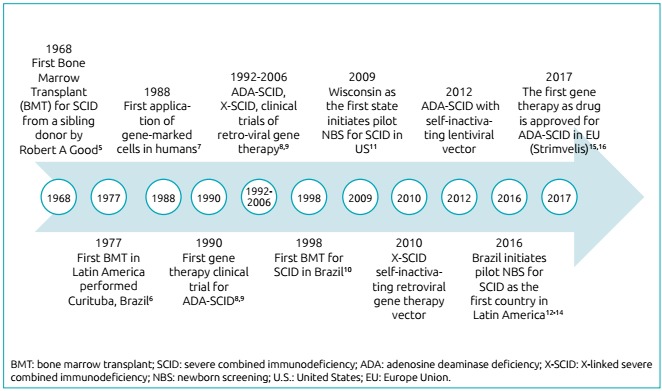
Timeline of severe combined immunodeficiency therapy.

Since the discovery of SCID in the 1960s, two major breakthroughs in treatment have re-defined clinical outcomes(Figure1).^5^ ^,^ ^6^ ^,^ ^7^ ^,^ ^8^ ^,^ ^9^ ^,^ ^10^ ^,^ ^11^ ^,^ ^12^ ^,^ ^13^ ^,^ ^14^ ^,^ ^15^ ^,^ ^16^

- bone marrow transplant (BMT) of healthy donor hematopoietic stem cells to SCID patients was introduced in 1968 in the United States.^17^ If successful, this approach can fully restore a normal immune system-T, B and natural killer (NK) cells;
- gene therapy was introduced in 1990.^18^ Throughthis process, the abnormal gene can be corrected in the patient’s own hematopoietic stem cell by viral transfer of the normal gene and, therefore, donor cells are not needed. This therapy has been implemented for two variants of SCID: adenosine deaminase deficiency (ADA-SCID) and X-linked SCID with IL2RGmutation.

Despite these therapeutic developments, many SCID patients are not being diagnosed early enough or are unable to gain access to the cited treatments. As expected, SCID is difficult to detect clinically in the asymptomatic period, unless the patient presents family history of SCID. Thus, the efficacy and optimal utilization of treatment is rooted in early detection of the disease with NBS. Ideally, SCID patients identified by NBS receive treatment before infection occurs, which greatly increases survival outcomes.^19^

### Implementation of NBS for SCID in the United States

Most patients with SCID will present severe naïve T-cell lymphopenia secondary to impaired T-cell development in the thymus.^20^ ^,^ ^21^ The United States is pioneering in implementation of SCID NBS, with an assay based on the detection of early abnormal T-cell development via T-cell receptor excision circles (TRECs). TRECs are generated during the process of T-cell receptor gene rearrangement in T-cell precursors in the thymus. Therefore, TRECs are enriched in the new immigrant naïve T-cells leaving the thymus. AsT-cells get activated and proliferate, they will not propagate TRECs. Therefore, activated cells will have low levels TRECs. Thus, TRECs are an indirect measure of naïve T-cells and thymic function. The assay was originally designed to assess remnant thymic function in peripheral blood of patients infected by human immunodeficiency virus (HIV) with T-cell lymphopenia.^22^ Chan and Puck have applied this assay first for evaluation of patients with SCID.^23^ ForNBS for SCID, the detection and quantification of TRECs are accomplished through extraction and amplification of deoxyribonucleic acid (DNA) from Guthrie cards obtained from infants around birth.

B-cell development can also be affected in several types of SCID. In addition to TRECs, a DNA-based assay has been developed to detect B-cell immunoglobulin light chain kappa receptor chain excision circle (KRECs). The absence of KRECs reflects abnormal B-cell development in the bone marrow and can accompany abnormal TRECs in forms of SCID that affect gene rearrangements, such as recombination activating gene (RAG) deficiency and components of the non-homologous end-joining complex (Table 1).^24^

**Table 1:** Genetic background of severe combined immunodeficiency (SCID) listed by immunological phenotype.

Immunological Phenotype	Gene Product	
T-B-NK+	DCLRE1 (ARTEMIS)	V(D)J recombination
	DNAPKcs	
	LIG4	
	PGM3	
	RAG1, RAG2	
	XLF (NHEJ1, Cernunnos)	
T-B+NK+	CD3δ	
	CORO1A	
	IL-7R	
	FOXN1	
	2q11 deletion (full DeGeorgesyndrome)	
	TBX1	
	LAT	
T-B+NK-	IL2RG “common γ chain	
	JAK3 Janus kinase 3	
	PNP	
T-B-NK-	ADA	
	AK2	
T-B-/+NK+/low	CD45	
T-B+NK+/low	RPP25 (RMRP)	
T+B-NK-	Hoyeraal-Hreidarsson Syndrome DKC1 (dyskeratin), TERT, TINF2, DCLRE1B (Apollo)	

T-B-NK+ Immunological Phenotype: DCLRE1: DNA cross-link repair 1C (artemis); DNA-PKcs: DNA-dependent protein kinase, catalytic subunit; LIG4: DNA ligase IV; XLF: XRCC4-like factor (Cernunnos) or NHEJ1: non-homologous end-joining factor; RAG1: recombination activating gene 1; RAG2: recombination activating gene 2; PMG3:phosphoglucomutase 3. T-B+NK+ Immunological Phenotype: CD3δ: cluster of differentiation 3 delta chain; CORO1A: coronin-1A; IL-7R: interleukin-7 receptor; FOXN1:forkhead box N1; 22q11.2 deletion (Full DiGeorge Syndrome); TBX1: T-box 1; LAT: linker for activation of T-cells; T-B+NK- Immunological Phenotype. IL2RG: interleukin 2 receptor subunit gamma (“common γ chain”); JAK3: Janus kinase 3; PNP: purine nucleoside phosphorylase; ADA: adenosine deaminase deficiency; AK2: adenylate kinase 2; CD45:cluster of differentiation (leukocyte common antigen); RMRP:RNA component of mitochondrial RNA processing endoribonuclease; DKC1: dyskerin pseudouridine synthase 1; TERT:Telomerase reverse transcriptase; TINF2: TERF1-interacting nuclear factor 2; DCLRE1B: DNA cross-link repair 1B protein (apollo).

Every country has different considerations regarding the inclusion of SCID on NBS panels. We believe that NBS for SCID should be implemented globally, which requires international efforts due to disparities in healthcare. In the United States, a disease must meet the following criteria to be considered for inclusion on the NBS panel:^25^ ^,^ ^26^

- minimum incidence of 1:100,000;
- fatality without treatment;
- improvement of outcomes with early treatment;
- development of a robust feasible test;
- a reasonable false positive rate;
- early presentation of disease.

**Figure 2:**
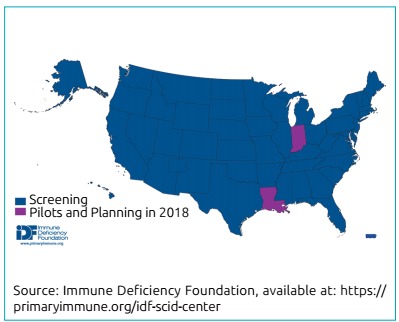
Severe combined immunodeficiency newborn screening implementation worldwide as of August2018.^30^

The US Secretary’s Advisory Committee on Heritable Disorders in Newborns and Children (SACHDNC) recommends the list of disorders to be screened by NBS. To date, 34congenital disorders have been added to the Recommended Uniform Screening Panel, and SCID was added in 2009.^27^ ^,^ ^28^ However, since the implementation of SCID NBS depends on state legislatures, the implementation time is variable across the United States. Since the first pilot program began in Wisconsin in 2009, 47 of the 50 states, the District of Columbia and Puerto Rico have sequentially implemented or have committed to implement SCID NBS (Jeffrey Modell Foundation;Figure 2).^11^ ^,^ ^29^ ^,^ ^30^

Apart from the United States, SCID NBS programs have implemented natiowide in Israel, Norway, and Taiwan, and in parts of Canada, and Spain (Figure 2), according to the Jeffrey Modell Foundation. In other countries, pilot screening programs have been initiated in France (2006), Germany (2010), Sweden (2013), United Kingdom (2013), and Belgium (2012).^13^ ^,^ ^31^ ^,^ ^32^ ^,^ ^33^ ^,^ ^34^ ^,^ ^35^ ^,^ ^36^ ^,^ ^37^ Routine, nationwide implementation of pilot or regional NBS programs have been limited by financial and legislative issues.

The false positive rate for detection of SCID by the TREC assay is high, as other conditions with naïve T-cell lymphopenia may test positive (Table 2). Therefore, thorough follow-up with secondary confirmation methods such as flow cytometry for naïve T-cell subsets and functional assays are required (see ahead). Once SCID variants are excluded, patients with T-cell lymphopenia may tolerate vaccinations without complications.^38^

**Table 2:** Alphabetized list of conditions and/or genetic defects associated with T cell lymphopenia identified by newborn screening (NBS) for severe combined immunodeficiency (SCID).

ATM (ataxia telangiectasia)	DOCK8	Moesin deficiency	SMARCAL1
BCL10	IKBKB,	MTHFD1	STAT5B
BLC11B	IKBK2	NOLA2	STIM1
CARD11	IL-21R	NOLA3	STK4 (MST1)
CD3e	ITK	ORAI1 (CRACM1)	TAP1/TAP2/tapasin
CD3g	Jakobsen	PCFT	TCN2
CD3z	LCK/p56	PRKDC	TCRa
CD8A	MAGT1 (X-MEN syndrome)	PTPRC	Trisomy 21 (Down syndrome
CHARGE (CHD67)	MALT-1	RAC2	TTC7A
DOCK2	MHCII*	RHOH	UNC119
			ZAP70

*alias CIITA, RFXANK, RFX5, RFXAP.

### Confirmatory testing and treatment following positive NBS for SCID

Once a patient is screened positive by NBS for SCID, the diagnosis needs to be confirmed with laboratory testing. Thesetests assess the immune system of the patient, including the lymphocyte count with subset analysis of naïve and memory T-cells, B and NK cells and lymphocyte proliferation studies. Low counts of autologous T-cells (<300 cells / µl) with low T-cell proliferation (<10% of lower level of normal) upon stimulation with phytohemagglutinin (PHA) are currently the diagnostic criteria of classical SCID.^39^ Thereare additional SCID variants (*e.g.*, leaky SCID, Omenn syndrome and variant SCID) that present higher counts of autologous T-cells (300-1,000 cells / µl) with improved, but low T-cell proliferation (10-30% of lower level of normal lymphocyte proliferation with PHA).^39^ In addition, it is also recommended that naïve T-cell count and fraction are determined, as it reflects well the abnormal thymic activity and T-cell development.

While being prepared for hematopoietic stem cell transplantation (HSCT), the patient must be isolated at home or in the hospital to avoid exposure to infectious agents. Currently, there is no consensus on whether asymptomatic patients should be hospitalized. As patients may contract infections, strategies need to be developed for monitoring of infections and avoiding them by use of prophylactic antimicrobials and other interventions. About 42% of SCID infants identified by NBS develop infections prior to receiving definitive therapy.^40^ Cytomegalovirus (CMV) is serious and life-threatening in SCID infants and is associated with increased risk for graft *vs*. host disease (GVHD) in patients receiving allogeneic transplantation. CMV is transmissible from a mother’s birth canal and/or breast milk. Therefore,infants with SCID whose mothers are seropositive should not be breastfed. The indication for prophylactic treatment for CMV is debated as it can cause neutropenia.^41^ ^,^ ^42^ While waiting for transplant, bridging therapies include immunoglobulin replacement, antimicrobial (fungal, viral and bacterial) treatments and in specific cases enzyme replacement therapy for SCID with adenosine deaminase (ADA) deficiency (Table 3).^43^

**Table 3:** Recommended infectious disease prophylaxis for newborns with suspected of severe combined immunodeficiency (SCID).

Prophylaxis in Newborn	Drug	Time of initiation	Alternatives	Comments
PCP	TMP-SMX orally (5 mg TMP/kg once a day for 2 consecutive days weekly)	1 month old	Atovaquone orally (30 mg/kg once a day)	Verify that bilirubin is <2X’s upper limit of normal before starting. Monitor ALT, AST, and bilirubin every 2-4 weeks
HSV	Acyclovir orally (20 mg/kg/dose 3 times a day)	At first visit		Follow BUN and creatinine every 2-4 weeks
Respiratory syncytial virus	Palivizumab (15 mg/kg/ I.M.)	1 month old		Given during peak RSV season, typically November-March in the northern hemisphere
General (bacterial/viral)	IVIG (0.4-0.5 g/kg every month) or SCIG	1 month old		Monitor troughs monthly and maintain Ig>600 mg/dl; Based on subcutaneous fat and body surface area to volume of medication administered, could consider SCIG in select patients
Fungal	Fluconazole (6 mg/kg once daily)	1 month old		Follow AST, ALT, and bilirubin every 2-4 weeks
In family members or close contacts				
Influenza	Inactivated influenza vaccine	Seasonally		
Pertussis	Tdap vaccine	Per routine childhood vaccinations		One booster for adolescents (11-12 years age); adults 19-64 years age and adults >65 years age

PCP: pneumocystis carinii pneumonia; HSV: herpes simplex virus; TMP-SMX: trimethoprim / sulfamethoxazole; IVIG: intravenous immunoglobulin; SCIG: subcutaneous immunoglobulin; ALT: alanine aminotransferase; AST: aspartate aminotransferase; BUN: blood urea nitrogen; RSV: respiratory syncytial virus; SCIG: subcutaneous immunoglobulin. Source: Thakar etal.^23^

During bridge therapy, the patient waits for the optimal setting of HSCT from a full human leukocyte antigens (HLA)-matched sibling or unrelated donor. If not available, most SCID patients receive haploidentical stem cells from parents (haploidentical transplant), especially if T-cells are absent, and, therefore, the likelihood of GVHD is lower. For patients without an optimal donor, autologous HSCT gene therapy (HSCT-GT) may be an option and has been highly successful. In fact, HSCT-GT is recommended as an equal first line therapy for ADA-deficiency and is advantageous for avoiding risk of severe GVHD.^44^

Haploidentical donors increase the risk for GVHD. Therefore, SCID patients, especially those with T-cells, may require conditioning.^40^ With reduced intensity conditioning, the bone marrow environment is optimized for engraftment of donor hematopoietic stem cells. There is a debate regarding the earliest time when conditioning can be safely used. Some centers have a long track record of no conditioning in infancy even at the expense of partial immune reconstitution with low B-cell function and the need for lifelong immunoglobulin replacement therapy. Depending on the underlying genetic defect, outcomes may be improved by using conditioning regardless of age, for example in SCID patients with hypomorphic RAG deficiency or DNA repair (non-homologous end joining) defects (Table 1).^45^

### Obstacles to NBS for SCID internationally

There is an unmet need for early detection of SCID patients globally, including in developing countries such as Brazil. Early live vaccinations and exposure to a wide variety of infectious agents may lead to clinical infections that worsen transplant outcomes and increase healthcare costs for management of these patients.^46^ Therefore, the outcome in countries without NBS for SCID remains sub-optimal with increased morbidity and mortality despite advances in therapy. Theinitiation of SCID NBS faces challenges in Brazil. National efforts for SCID NBS should be supported by several centers with high diagnostic and transplant expertise in SCID. These centers should be evenly dispersed across the country to ensure access and coverage. Ideally, these centers should also prioritize and allocate resources for the routine care of SCID patients, including beds, organization of an inpatient and outpatient clinical care team and development of hospital protocols.

The introduction of the Guthrie card in 1963 has resulted in the widespread use of this simple but universal NBS device that is available globally. Blood spots on the card, obtained from a heel prick, can be analyzed to detect rare genetic, metabolic, and endocrine diseases. DNA remains stable on this card and can be a reliable source of detection of TRECs. NBS began in Brazil in 1976, and, from 2001 to 2005, about 13 million newborns were screened, with coverage increasing from 55 (in 1976) to 80.2% (in 2005).^12^ Despite these advancements in national NBS implementation, Brazil is still working to fully incorporate SCID into their list of nationally screened diseases. Over the last several years, academic research projects through the University of São Paulo (USP), Federal University of São Paulo (UNIFESP) and the Jeffrey Modell Foundation Diagnostic and Research Center of São Paulo have implemented two pilot programs for NBS in Brazil. The first Brazilian SCID NBS pilot launched in 2016 and screened 8,715 newborns using the TRECs assay.^13^ The second pilot launched in 2017 and screened 6,881 newborns using both the TRECs and KRECs assays, with sample collections in several metropolitan areas in the São Paulo region.^14^ Both of these pilot programs confirmed that SCID NBS assay is reliable and feasible for future implementation on a national scale in Brazil.

Without effective infrastructure for early HSCT, there is only partial value in NBS for SCID. Yet, many countries among Central and Latin America are leading efforts to improve treatment for SCID. In 1976, Colombia was the first country to conduct a HSCT. Similarly, since that time Brazil has established infrastructure to provide many key therapies for SCID. In 1979, the first organized Brazilian HSCT program was established in the city of Curitiba, in state of Paraná. To improve the HLA-matching for donor and recipient, HSCT program initially began with sibling matched donors and evolved to alternative donor transplantation in 1995. With the introduction of post-transplantation cyclophosphamide to prevent GVHD, haploidentical transplantation was initiated. Thefirst HSCT for patients with SCID were conducted in Central and Latin America in 1985 in Costa Rica and in 1998 in Brazil, respectively.^10^ For a population of over 200 million inhabitants in Brazil, there are close to a hundred BMT medical units. Ofapproximately 3,000 HSCT performed in the period of 1979-2018 for various health conditions in Curitiba, 90% of these were allogenic. This magnitude of population and growing level of expertise underscores the importance of screening program for SCID in Brazil.

Families are getting smaller in Brazil, as in most developed countries, thereby decreasing the chance of finding a sibling donor. Brazilian BMT units are unable to do haploidentical transplant with T-cell depletion, and thus use post-HSCT treatment with cyclophosphamide to remove donor T-cells is needed to reduce the risk of GVHD. Brazil has developed a donor registry entitled *Registro Nacional de Doadores Voluntários de Medula Óssea* (REDOME), that currently has more than four million donors registered. Therefore, it is the third largest bone marrow volunteer donor registry in the world. In addition, there are 11 public cord blood banks in Brazil, even though cord blood transplantation is decreasing after the emergence of HSCT treatment with post-transplant cyclophosphamide. Unfortunately, despite the ample infrastructure for HSCT technology, there are inadequate numbers of personnel trained in the specialized HSCT for SCID patients in Brazil and Latin America.

Since the initial pilot studies, Brazil has reached the fourth phase of implementation of SCID NBS within the country. Experts in immunology advocate on all levels for the implementation of NBS for SCID and other primary immunodeficiencies during the first year of life as it would decrease clinical costs and improve public health. In fact, the Brazilian Society of Allergy and Immunology is currently applying to incorporate the NBS for SCID and possibly other primary immune deficiencies (PIDs) in the national screening program together with other rare diseases. This request is pending approval andfunding.^13^ ^,^ ^14^

To optimize the implementation of these advancements, it is essential to ensure that patients have access confirmatory services for the diagnosis of SCID after positive NBS. These diagnostic services include machinery to quantify lymphocytes subpopulations (T and naïve T-cells) and function (lymphocyte proliferation assays). Unfortunately, these tests are not universally available, but only in large academic research centers.

### Economic impact of NBS for SCID

From a long-term economic perspective, screening programs and treatments for early diagnosis of asymptomatic SCID patients are less expensive than providing healthcare to a child that has a delayed diagnosis and complicating infections before definitive therapies are initiated.

Globally, short-term implementation costs may be a barrier to adding SCID to NBS panels, but it could be justified by the cost difference between transplanting a child above and below 3.5 months of age with or without infections. For example, in the United States in 2014, the mean total charges for late transplantation for SCID per patient were four times greater than early treatment ($ 1.43 million *vs*. $ 365,785 respectively) without consideration of the potential need for intensive care services.^47^ Thecost-effectiveness of early treatment for SCID provided strong economic justification for the addition of SCID screening to NBS programs in all states in the United States by 2018. Brazilhas not performed a thorough cost-benefit analysis of the cost of SCID NBS and treatment before or after the onset of infections.^14^

Making the cost of SCID NBS comparable to or less than that for treatment on a population level will facilitate government approval of nationwide SCID NBS programs. Healthcare cost for SCID treatment, including HSCT, are lower in Europe^48^ and in the developing world than in the United States. Therefore it is less expensive for these countries to treat SCID once symptoms present. Thus, implementing countrywide SCID NBS programs may be a lesser healthcare priority in most of Europe and in developing countries than in the US. However, not implementing these programs results in greater infant mortality and morbidity;^32^ ^,^ ^47^ failure to consider this fact leads to overestimating the economic cost/benefit ratio of NBS. Further, recent modifications to the NBS assay can lower its costs. TheTREC NBS assay for SCID costs approximately $5pepatient in the United States.^47^ ^,^ ^48^ ^,^ ^49^ A German study lowered the cost of SCID NBS to € 2 per sample ($ 2.33) by reducing the sample size used for testing, devising a more efficient DNA extraction technique and using internal controls selectively.^50^ The reduced cost of the new SCID NBS method, the marked increase in cost of late *versus* early SCID treatment, and the long-term monetary value of saving lives with early screening and treatment^51^ are strong economic rationales, besides ethical justification, for considering SCID NBS throughout Brazil and the rest of the world where it is not performed.

### Impact of NBS on SCID incidence and patient survival

SCID NBS saves lives. For example, a multi-site study conducted by the Primary Immune Deficiency Treatment Consortium found that infants not tested until symptoms presented had a 58% survival rate, compared to 85% survival for infants tested at birth.^40^ The implementation of SCID NBS on the Recommended Uniform Screening Panel has dramatically changed the clinical presentation of SCID in the United States. Analysis of screening of three million newborns for SCID after the initiation of SCID NBS confirmed a higher-than-expected prevalence of 1:58,000, increasing from 1:100,000 in 2009 prior to NBS. In the United States, X-linked SCID remains the most common variant among SCID patients. However, its relative frequency has decreased from 46 to 19% and recombinase activating gene (RAG1/2) deficiency is becoming dominant in leaky SCID variants.^41^ ^,^ ^52^ Pathogenic variants are now the norm. Furthermore, the frequency of SCID across racial and ethnic groups is increasing following implementation of SCID NBS. There is also founder mutation penetrance in communities with frequency up to 1:2,000, found in communities of Somali, Amish, Mennonite, Navajo Indians and Irish Traveler descent.^53^ ^,^ ^54^ ^,^ ^55^ ^,^ ^56^

To broaden newborn screening for immunodeficiency, a new program, “Following Infants with Low Lymphocytes” (FILL), has been organized by the Clinical Immunology Society (CIS) and the United States Immunodeficiency Network (USIDNET). This program is designed to track the diagnoses and outcomes of non-SCID patients identified with T-lymphopenia in the NBS program^57^ (Table 2).

## CONCLUSION

Early diagnosis of SCID is feasible by using a Guthrie screening card shortly after birth. Although the method is relatively inexpensive, it requires centralized laboratory testing and a network of clinical immunologists to confirm the clinical and genetic diagnosis, and a BMT team to perform HSCT with optimal timing and selection of donor and conditioning regimen. With international effort addressing the challenges and solutions to managing SCID in newborns, the dire consequences of this disease can be thwarted, thus relieving the tremendous fiscal, social, and emotional burden of affected children and families worldwide.
