# ERRATUM

**DOI:** 10.1590/1984-0462/;2019;37;2;00020erratum

**Published:** 2020-04-30

In the manuscript “In time: The value and global implications of newborn screening for severe combined immunodeficiency”, DOI: 10.1590/1984-0462/;2018;36;4;00020, published in the Rev Paul Pediatr. 2018;36(4):388-397:

Page 388:

Where it reads:

Cristina Meehana

Jolan Walter

It should read:

Cristina A. Meehan

Jolan E. Walter

Page 389:

Page 389: Where it reads:

**Figure 1:**
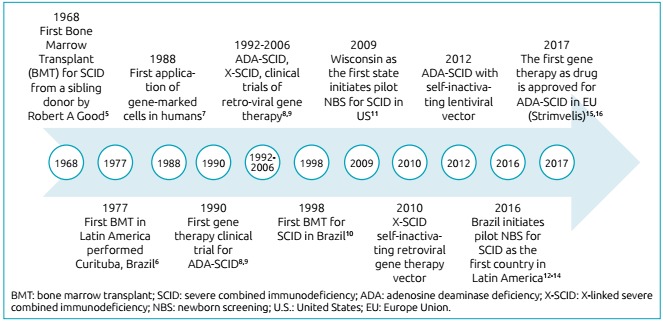
Timeline of severe combined immunodeficiency therapy.

It should read:

**Figure 1:**
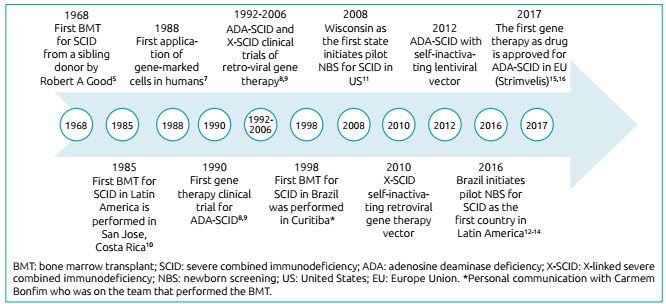
History of severe combined immunodeficiency therapy.

Page 390, first column:

Where it reads:

**Table 1:** Genetic background of severe combined immunodeficiency (SCID) listed by immunological phenotype.

Immunological Phenotype	Gene Product	
T-B-NK+	DCLRE1 (ARTEMIS)	}V(D)J recombination
	DNAPKcs	
	LIG4	
	PGM3	
	RAG1, RAG2	
	XLF (NHEJ1, Cernunnos)	
T-B+NK+	CD3δ	
	CORO1A	
	IL-7R	
	FOXN1	
	2q11 deletion (full DeGeorge syndrome)	
	TBX1	
	LAT	
T-B+NK-	IL2RG “common γ chain	
	JAK3 Janus kinase 3	
	PNP	
T-B-NK-	ADA	
	AK2	
T-B-/+NK+/low	CD45	
T-B+NK+/low	RPP25 (RMRP)	
T+B-NK-	Hoyeraal-Hreidarsson Syndrome DKC1 (dyskeratin), TERT, TINF2, DCLRE1B (Apollo)	

T-B-NK+ Immunological Phenotype: DCLRE1: DNA cross-link repair 1C (artemis); DNA-PKcs: DNA-dependent protein kinase, catalytic subunit; LIG4: DNA ligase IV; XLF: XRCC4-like factor (Cernunnos) or NHEJ1: non-homologous end-joining factor; RAG1: recombination activating gene 1; RAG2: recombination activating gene 2; PMG3: phosphoglucomutase 3.

T-B+NK+ Immunological Phenotype: CD3δ: cluster of differentiation 3 delta chain; CORO1A: coronin-1A; IL-7R: interleukin-7 receptor; FOXN1: forkhead box N1; 22q11.2 deletion (Full DiGeorge Syndrome); TBX1: T-box 1; LAT: linker for activation of T-cells; T-B+NK- Immunological Phenotype.

IL2RG: interleukin 2 receptor subunit gamma (“common γ chain”); JAK3: Janus kinase 3; PNP: purine nucleoside phosphorylase; ADA: adenosine deaminase deficiency; AK2: adenylate kinase 2; CD45: cluster of differentiation (leukocyte common antigen); RMRP: RNA component of mitochondrial RNA processing endoribonuclease; DKC1: dyskerin pseudouridine synthase 1; TERT: Telomerase reverse transcriptase; TINF2: TERF1-interacting nuclear factor 2; DCLRE1B: DNA cross-link repair 1B protein (apollo).

It should read:

**Table 1:** Genetic background of severe combined immunodeficiency (SCID) listed by immunological phenotype.

Immunological Phenotype	Gene Product	
T-B-NK+	DCLRE1C (ARTEMIS)	}V(D)J recombination
	DNA-PKcs	
	LIG4	
	PGM3	
	RAG1, RAG2	
	XLF (NHEJ1, Cernunnos)	
T-B+NK+	CD3δ	
	CORO1A	
	IL-7R	
	FOXN1	
	22q11.2 deletion (full DiGeorge syndrome)	
	TBX1	
	LAT	
T-B-NK+	IL2RG	
	JAK3	
T-B-NK-	PNP	
	ADA	
	AK2	
T-B-/+NK+/low	CD45	
T-B+NK+/low	RPP25 (RMRP)	
T+B-NK-	Hoyeraal-Hreidarsson Syndrome DKC1 (dyskerin), TERT, TINF2, DCLRE1B (Apollo)	

T-B-NK+: T and B cell negative, natural killer cell positive; DCLRE1: DNA cross-link repair 1C (artemis); DNA-PKcs: DNA-dependent protein kinase, catalytic subunit; LIG4: DNA ligase IV; XLF: XRCC4-like factor (Cernunnos) or NHEJ1: non-homologous end-joining factor; RAG1: recombination activating gene 1; RAG2: recombination activating gene 2; PMG3: phosphoglucomutase 3.

CD3δ: cluster of differentiation 3 delta chain; CORO1A: coronin-1A; IL-7R: interleukin-7 receptor; FOXN1: forkhead box N1; 22q11.2 deletion (Full DiGeorge Syndrome); TBX1: T-box 1; LAT: linker for activation of T cells; IL2RG: interleukin 2 receptor subunit gamma (“common γ chain”); JAK3: Janus kinase 3; PNP: purine nucleoside phosphorylase;

ADA: adenosine deaminase deficiency; AK2: adenylate kinase 2; CD45: cluster of differentiation (leukocyte common antigen 45); RMRP: RNA component of mitochondrial RNA processing endoribonuclease; DKC1: dyskerin pseudouridine synthase 1; TERT: Telomerase reverse transcriptase; TINF2: TERF1-interacting nuclear factor 2; DCLRE1B: DNA cross-link repair 1B protein (Apollo).

Page 390, second column:

Where it reads:

However, since the implementation of SCID NBS depends on state legislatures, the implementation time is variable across the United States. Since the first pilot program began in Wisconsin in 2009, 47 of the 50 states, the District of Columbia and Puerto Rico have sequentially implemented or have committed to implement SCID NBS (Jeffrey Modell Foundation; Figure 2).^11,29,30^

It should read:

Since the first pilot program began in Wisconsin in 2008, all 50 states, the District of Columbia and Puerto Rico have sequentially implemented SCID NBS (Immune Deficiency Foundation, Jeffrey Modell Foundation; Figure 2A)^11,29,30^

Where it reads:

**Figure 2:**
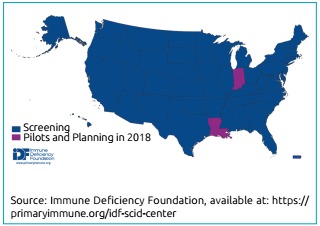
Severe combined immunodeficiency newborn screening implementation worldwide as of August 2018.^30^

It should read:

**Figure 2:**
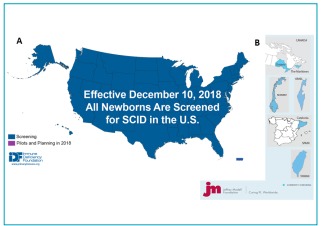
Severe combined immunodeficiency newborn screening implementation in the United States^*^ (A) and worldwide^30^ (B) by December 2018.

*Immune Deficiency Foundation. SCID Newborn Screening: Current Status of Implementation Map [Internet]. December 2018 [cited on December 21, 2018]. Available at: https://primaryimmune.org/idf-advocacy-center/idf-scid-newborn-screening-campaign.

Page 394,

Where it reads:

[...] Analysis of screening of three million newborns for SCID after the initiation of SCID NBS confirmed a higher-than-expected prevalence of 1:58,000, increasing from 1:100,000 in 2009 prior to NBS. [...]

It should read:

[...] Analysis of screening of three million newborns for SCID after the initiation of SCID NBS confirmed a higher-than-expected prevalence of 1:58,000, increasing from 1:100,000 in 2008 prior to NBS.^25^ [...]

Where it reads:

We thank and acknowledge Dr. Jane Carver from the University of South Florida, for their assistance in the editing of this document.

It should read:

We thank and acknowledge Dr. Jane Carver from theUniversity of South Florida for assistance in editing this document.

Page 395,

Where it reads:

30. Jeffrey Modell Foundation. Newborn screening for SCID. Update on the implementation of newborn screening for SCID in the United States [Internet]. August 2018 [cited on Sept. 25, 2018]. Available at: http://www.info4pi.org/ town-hall/newborn-screening

It should read:

30. Adapted from Jeffrey Modell Foundation. Newborn screening for SCID. Update on the implementation of newborn screening for SCID in the United States [Internet]. December 2018 [cited on January 13, 2019]. Available at: http://www.info4pi.org/ town-hall/newborn-screening.

